# Time‐Restricted Feeding Promotes Longevity and Gut Health Without Fitness Trade‐Offs

**DOI:** 10.1096/fj.202500875R

**Published:** 2025-05-14

**Authors:** Ann‐Cathrin Hofacker, Mirjam Knop, Susanne Krauss‐Etschmann, Thomas Roeder

**Affiliations:** ^1^ Zoology, Department of Molecular Physiology Christian Albrechts University Kiel Kiel Germany; ^2^ Research Center Borstel, Priority Research Area Chronic Lung Diseases, Early Life Origins of CLD Borstel Germany; ^3^ Department of Medicine Christian Albrechts University Kiel Germany; ^4^ German Center for Lung Research Airway Research Center North Kiel/Borstel Germany

## Abstract

Time‐restricted feeding (TRF), a dietary intervention involving daily fasting periods, has been associated with metabolic benefits; however, its long‐term physiological impact remains unclear. Using *Drosophila melanogaster* as a model, we investigated the effects of a 16:8 TRF regimen on lifespan, reproductive output, gut health, and microbiota composition. TRF significantly extended lifespan, even when applied only during early adulthood. Notably, this longevity benefit occurred without compromising reproductive fitness, as measured by female fecundity in life's most crucial reproductive phase. TRF promoted gut homeostasis in aged flies by reducing intestinal stem cell proliferation and enhancing epithelial barrier integrity. Furthermore, TRF induced a shift in microbiota composition, increasing the prevalence of gram‐negative bacterial taxa. These results show that even short‐term TRF interventions at a young age can have long‐term physiological benefits. Metabolic reprogramming or increased autophagy are the most likely mechanisms mediating the health‐promoting effects of this type of nutritional intervention. TRF is an effective, non‐invasive strategy for promoting healthy longevity without significant adverse effects on other aspects of life.

## Graphical abstract

Short periods of time‐restricted feeding (TRF) applied in early‐life and lifelong TRF increased survival compared to *ad libitum* feeding. Noticeably, shorter TRF periods also increased lifespan, and health‐promoting effects of these interventions were not accompanied by reduced fecundity. Instead, improved gut integrity and healthy aging were observed, demonstrating the beneficial effects of TRF on numerous metabolic parameters.

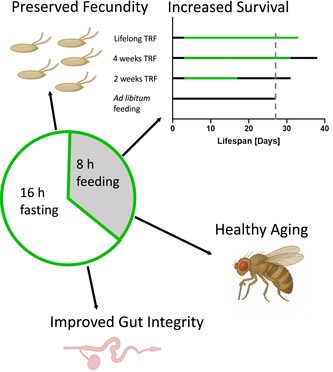

## 1 Introduction

Nutritional habits play a central role in the development and progression of metabolic disorders, including obesity, type 2 diabetes, and cardiovascular diseases [1, 2, 3]. While certain dietary patterns are associated with increased health risks, specific nutritional interventions, such as caloric restriction (CR) and dietary protein restriction (DR), have demonstrated protective effects across species, contributing to improved metabolic function, delayed onset of chronic diseases, and an extended lifespan [4, 5, 6, 7, 8, 9].

Beyond nutrient composition and caloric intake, recent evidence suggests that the timing of food consumption is also a critical factor influencing metabolic homeostasis. Time‐restricted feeding (TRF), or time‐restricted eating (TRE) in humans, limits food intake to a defined daily window without necessarily reducing caloric intake. Protocols ranging from 12‐h to more stringent 16‐h fasting intervals have been evaluated, with 16:8 TRF (16 h fasting, 8 h feeding) consistently associated with improved glucose tolerance, reduced insulin resistance, and lowered blood pressure [10, 11, 12, 13, 14, 15, 16, 17, 18]. Mechanistically, the beneficial effects of TRF are mediated by induced circadian autophagy and metabolic reprogramming [19, 20].

*Drosophila melanogaster* is a powerful model for probing the physiological and molecular effects of dietary interventions. With a short lifespan, conserved nutrient‐sensing pathways, and a tractable genetic toolkit, *Drosophila* enables quantitative analysis of how nutritional regimens influence aging, stress responses, and tissue integrity [21, 22, 23, 24]. Previous studies have shown that various forms of intermittent fasting, including diurnal TRF (12:12 cycles), can promote longevity, maintain gut barrier function, enhance mitochondrial function, and preserve circadian rhythm in flies [25, 26, 27, 28, 29, 30]. Yet, the specific effects of the widely used 16:8 TRF regimen remain largely uncharacterized in this model. This is particularly important because a 16:8 form of TRF may have more significant health effects, such as increased autophagy and metabolic reprogramming, than a 12:12 form of TRF.

Moreover, it remains unclear whether short‐term exposure to TRF, especially when applied early in life, can elicit long‐lasting physiological benefits, and whether such interventions incur costs to reproductive output, a common trade‐off in longevity studies. Given the similarities between *Drosophila* and mammalian metabolic and gastrointestinal systems [31, 32], this model provides a unique opportunity to explore these unresolved questions in a controlled and standardized setting and to test the hypothesis that 16:8 TRF exerts particularly positive effects on health and lifespan.

This study employed a 16:8 TRF paradigm in *Drosophila melanogaster* to investigate its effects on lifespan, reproductive fitness, gut homeostasis, and microbiota composition. We aimed to determine whether early‐life or intermittent TRF is sufficient to confer long‐term benefits and to evaluate potential trade‐offs between somatic maintenance and reproduction.

## 2 Material and Methods

### 2.1 Time‐Restricted Feeding and General Experimental Conditions

Time‐restricted feeding consisted of one feeding period and one fasting period each day (24‐h cycle). Flies had access to food for 8 h, beginning at Zeitgeber time (ZGT) 0 and concluding at ZGT 8. From ZGT 8 to ZGT 24, flies only had access to a water supply containing 1%–2% agar‐agar. Unless noted otherwise, the experiments were carried out in a climate chamber maintained at 25°C, 65% humidity, and a 12‐h light–dark cycle, with the flies being fed a rich medium [33]. TRF was implemented for 6 consecutive days, followed by a 24‐h period of *ad libitum* access. The control group had *ad libitum* access to food for the entire measurement period of the respective experiment.

### 2.2 Food Consumption

With minor alterations, food consumption was measured based on Shell et al. [34]. Flies were individually nurtured in 2 mL screw‐cap tubes for 8 to 14 days. TRF (2.1) was implemented by switching the lids of the screw‐cap tubes: at the beginning of the feeding periods, lids with blue‐colored food (75 μL of concentrated lab medium containing 2% erioglaucine disodium salt) were only lightly tightened on the tubes to allow airflow. Afterward, the lids were carefully removed and replaced with those containing 1%–2% agar‐agar for the subsequent starvation period. The lids of the control group remained unchanged throughout the day. All unused lids were stored at 8°C. After each 24‐h interval, the flies were individually transferred to a new 2 mL screw‐cap tube, and TRF was repeated. The empty tubes with excrement were kept at −20°C until analysis.

The analysis of the amount of consumed food was conducted as follows: 1 mL of Millipore water and three ceramic beads were added to each of the tubes, and the excrements were homogenized using a bead ruptor (OMNI Bead Ruptor 24, BiolabProducts, Bebensee, Germany) for 2 min at 3.25 m/s. The solution was centrifuged at 3000 *g* and 4°C for 3 min. 200 μL of the supernatants of each sample was pipetted in duplicates in a 96‐well plate, and the absorbance was measured at 630 nm wavelength. A standard was prepared by diluting 150 mg of the blue‐colored food medium in 1 mL of water, followed by homogenization and centrifugation of that sample. Afterward, a serial 1:2 dilution of the standard and a blank were pipetted in triplets in a 96‐well plate, and the absorbance at 630 nm was measured. The amount of food in every sample was calculated based on the standard curve and the blanks.

### 2.3 Survival

Flies were grouped into sets of 20 individuals and raised in fly vials. TRF (2.2.1) was administered to half of the flies. The remaining half was the control group and was placed into new vials containing CM‐medium only on Mondays, Wednesdays, and Fridays. Each time the flies were transferred into new vials, the numbers of dead and escaped flies were recorded until all the flies perished. To assess both short‐term and long‐term effects of TRF on lifespan, TRF was also applied for 2 and 4 weeks before resuming *ad libitum* access.

### 2.4 Starvation Resistance

The resistance to starvation was assessed after 1 and 2 weeks of TRF (2.2.1). Flies were maintained on 1.5% agar‐agar and monitored using the Zantiks device for 90 h. The point of death was identified as the first hour without any movement.

### 2.5 Fecundity

TRF (2.2.1) was applied for 1, 2, and 4 weeks before returning to *ad libitum* food access. The number of eggs laid within 24 h was counted manually using a microscope. This procedure was carried out over 1 week. Flies were placed on NM to more accurately determine the number of eggs across 10 replicates, each containing 3 flies.

### 2.6 Body Composition

Each biological replicate consisted of five flies that were treated with feeding restriction (2.2.1) for 1, 2, or 4 weeks, respectively. After the final feeding period (with *ad libitum* access for the treatment and control group for 24 h), the flies were numbed on ice, collected in 2 mL screw‐cap tubes, and the total weight of each replicate was measured [Balance (MXX‐412)]. Then, three beads and 1 mL of 0.05% Tween‐20, freshly diluted in PBS, were added, and the flies were homogenized using a ruptor (OMNI International, OMNI Bead Ruptor 24) for 2 min at 3.25 m/s. The samples were then heat‐inactivated for 5 min at 70°C and centrifuged at 3000 rpm for 3 min. Seven hundred microliters of the supernatants were transferred into new 2 mL screw‐cap tubes, and the samples were stored at −20°C until further analysis.

The body fat of *Drosophila* primarily consists of triglycerides (TAGs) stored in lipid droplets. The measurement was conducted as described previously [35, 36]. The samples were thawed, warmed to 37°C, briefly vortexed, and centrifuged at 3000 rpm for 3 min. Fifty microliters of the supernatants from each replicate and a blank containing 50 μL of PBT were pipetted in duplicate into a 96‐well plate. The trioleate standard was created by diluting 1 μL of a TAG aliquot in 450 μL of 0.05% PBT, followed by homogenization, heat inactivation, and centrifugation identical to the sample treatment. Subsequently, a serial 1:2 standard dilution was pipetted into a 96‐well plate and processed in the same manner as the samples. T0‐values were calculated by measuring the absorbance at 500 nm using a photometer (SYNERGY H1 microplate reader, BioTek). To initiate the reaction, 200 μL of a pre‐warmed (37°C) triglyceride solution (Triglyceride Reagent Kit, Pointe Scientific) was added to each sample and incubated for 10 min at 37°C with gentle shaking (200 rpm) on a plate shaker. Finally, the absorbance at 500 nm was measured again (T1‐values). The fat content calculation was based on the trioleate standard, the weight of the flies, and the absorbance measurements obtained at the start and after the reaction. According to the user's manual, the Pierce BCA Protein Assay Kit (Thermo Fisher Scientific) was used to measure the protein content. The samples were warmed to 37°C, briefly vortexed, and centrifuged at 3000 rpm for 3 min. Twenty‐five microliters of the supernatants from each replicate were pipetted in duplicate into a 96‐well plate. Additionally, a blank consisting of 25 μL PBT and 25 μL of each dilution of the albumin standard, prepared according to the user's manual of the Protein Assay Kit, was pipetted in duplicate. Afterward, 200 μL of the working reagent (Pierce BCA Protein Assay Kit, Thermo Fisher Scientific) was added to each well and mixed on a plate shaker (Shaking Incubator IS‐OS 20) for 30 s. Following incubation for 30 min at 37°C with mild shaking (200 rpm), the absorbance was measured at 562 nm using a plate reader (SYNERGY H1 microplate reader, BioTek). The calculation of the BSA content in the flies was based on the weight and absorbance measurements of the samples compared to the standard.

### 2.7 Zantiks Activity Monitoring

The activity of the flies was monitored for 3 days using the Zantiks device, following 1, 2, and 4 weeks of TRF (2.2.1), respectively. The flies were maintained in 100 μL of concentrated medium in a well of a 96‐well plate at 25°C with a 12‐h light–dark cycle. Movement per hour was calculated for each fly and analyzed using an R script.

### 2.8 Gut Integrity

After applying TRF (2.2.1) for 1, 2, and 4 weeks and subsequently providing *ad libitum* access, the flies were given a blue‐colored 5% sugar solution to assess gut integrity. They were kept in vials containing agar and filter paper soaked in the sugar solution for 24 h. Gut integrity was evaluated by counting the blue‐colored abdomens of the flies, indicating a loss of gut integrity and leakage of blue food into their bodies. These blue‐colored phenotypes of the flies are called “smurfs.”

### 2.9 Gut Dissection

The flies were numbed on ice and placed on a dissection plate containing PBS. 10 guts per biological replicate were dissected using forceps. The head was removed, and the rectal ampulla was cut off the cuticle. Then, the gut was carefully pulled in the anterior direction. The esophagus, crop, hindgut, and terminal parts of the Malpighian tubules were removed, and the remaining midgut was used for experiments.

### 2.10 Statistical Analysis

The primary collection and evaluation of the data were conducted using Excel and RStudio. Statistical analysis was performed with GraphPad Prism (version 9.5.0 for Windows, GraphPad Software, San Diego, California USA, www.graphpad.com). The data were tested for normality (normal Gaussian distribution) using the Shapiro–Wilk test. Normally distributed data were analyzed for statistical significance with paired or unpaired t‐tests, while non‐normally distributed data were assessed using nonparametric Mann–Whitney tests. Survival analyses were conducted using the Mantel‐Cox test (log‐rank). Results were considered statistically significant at a significance level of *α* = 0.05 (*p* < 0.05*; *p* < 0.01**; *p* < 0.001***; *p* < 0.0001****).

## 3 Results

**FIGURE 1:**
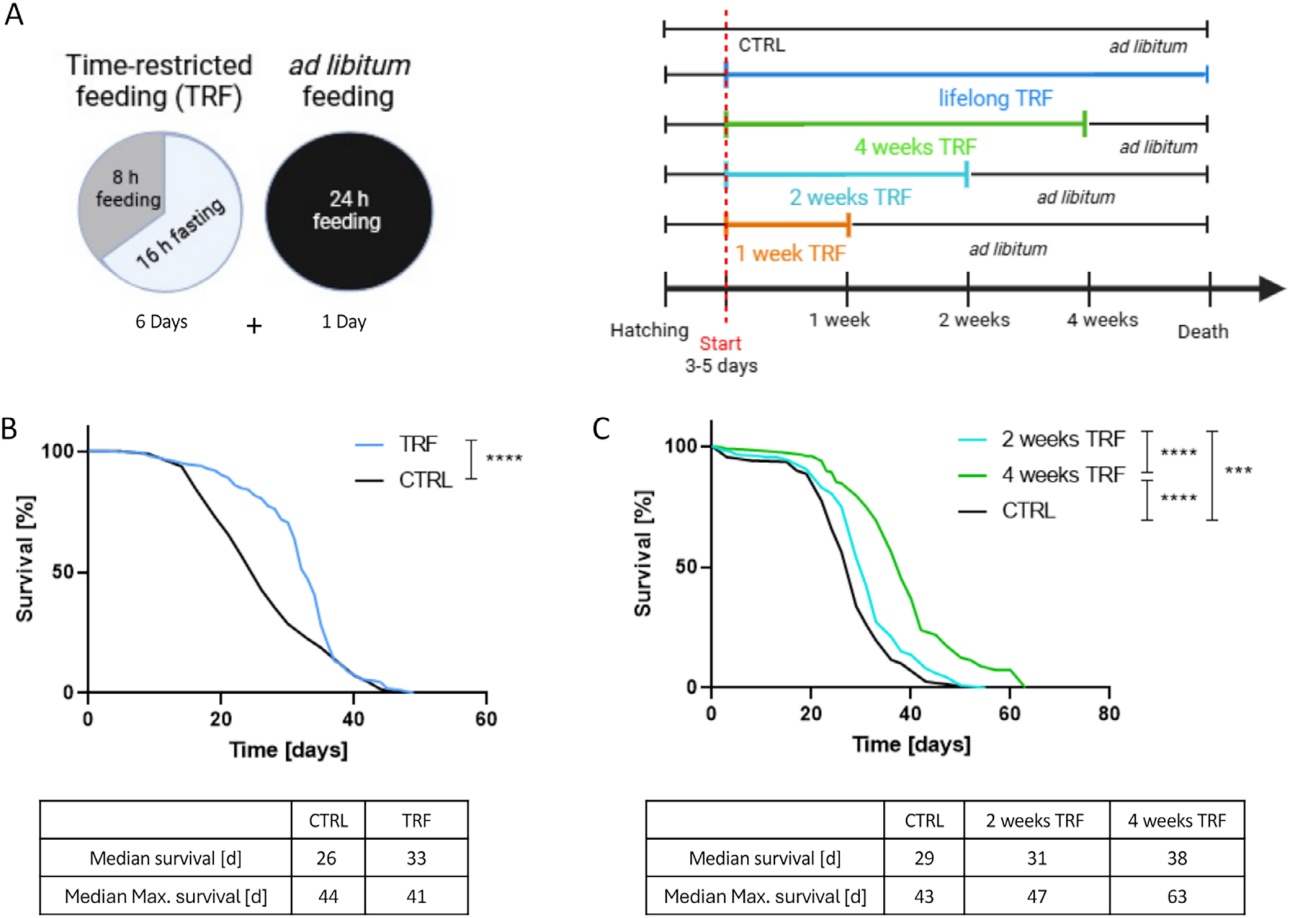
Schematic overview of experimental setup and lifespan under time‐restricted feeding in different intensities. (A) Representation of a week under experimental conditions containing 6 subsequent days of time‐restricted feeding and 1 day of *ad libitum* access (left). Overview of time‐restricted feeding applied in different intensities and their control (right). (B) Lifespan under lifelong time‐restricted feeding compared to a control with continuous *ad libitum* access. (C) Lifespan of two groups with time‐restricted feeding for 2 or 4 weeks and subsequent *ad libitum* access compared to a control with continuous feeding. *p* < 0.001***; *p* < 0.0001****; *n* = 200.

TRF constituted 8 h of access to food and 16 h of access to 1%–2% agar‐agar as water supply to prevent desiccation (Figure 1A). TRF was applied for different durations depending on the experimental setup. However, each week constituted 6 subsequent days with TRF followed by 1 day of *ad libitum* access (Figure 1A). The control group had continuous *ad libitum* access to food. Unless stated otherwise, mated female w^1118^ flies aged 3–5 days were used for the experiments. As external parameters such as population density, food composition, humidity, and temperature were observed to affect experimental outcomes independently, these were kept constant among all experiments unless explicitly noted [37, 38, 39].

### 3.1 TRF Prolongs Lifespan Significantly

To assess whether this specific form of TRF (16:8) affects lifespan, this nutritional intervention was applied to flies throughout their adulthood, and mortality was monitored until their death. Lifelong TRF prolonged the median lifespan of TRF flies by 26% compared to their control group. However, we observed no impact on the maximum lifespan with lifelong TRF (Figure 1B). Next, we implemented the TRF regime for defined periods before switching to continuous *ad libitum* access for their remaining lifetime (Figure 1C). 4 weeks of TRF, followed by a switch to continuous *ad libitum* feeding, significantly increased the lifespan of flies by 31%. Furthermore, this intervention also led to a significant increase in maximum lifespan, which was not observed with continuous TRF. Here, the maximal lifespan (median lifespan of the top 10% of animals with the longest lifespan) increased by 46.5%. Reducing the TRF period to 2 weeks still resulted in a significantly increased median lifespan, though the increase dropped to 7%, and the maximum lifespan extension to 9.3%. Thus, we observed a relationship between the length of the TRF period and its positive effect on lifespan.

### 3.2 TRF Enhances Lifespan Without Compromising Fecundity

**FIGURE 2:**
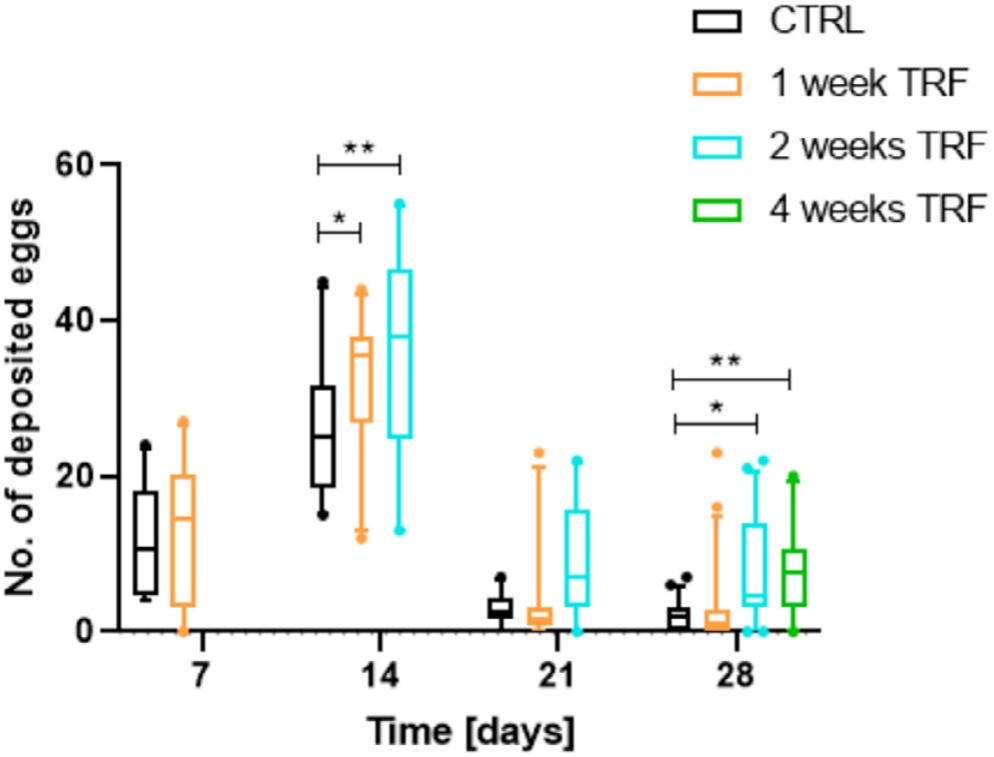
Fecundity measurements for four subsequent weeks with time‐restricted feeding in different intensities compared to a control group with continuous *ad libitum* access. *n* = 30, *p* < 0.05*, *p* < 0.01**, Box and whiskers represent mean and 10–90 percentile.

Since we observed an increased lifespan associated with different durations of TRF, we speculated that this might come at the expense of fitness, specifically fecundity. Therefore, we quantified fecundity after defined periods of TRF (1, 2, and 4 weeks) and compared these measurements to the fecundity of animals with continuous access to *ad libitum* feeding. We measured the number of eggs laid within 24 h, once a week for four consecutive weeks (Figure 2). A significant difference in fecundity was noted after 2 weeks under experimental conditions, as the number of laid eggs increased significantly by 18% (1‐week TRF) and 25% (2‐weeks TRF) compared to the respective control (means: CTRL = 26 eggs per fly, 1‐week TRF = 32 eggs per fly, 2 weeks TRF = 35 eggs per fly). After 3 weeks, an overall decreasing trend was observed, with no significant differences in the absolute number of laid eggs among the groups. However, after 4 weeks under experimental conditions, the number of laid eggs increased significantly for both 4 weeks of continuous TRF and 2 weeks of TRF compared to the control group and 1 week of TRF (means: CTRL = 2 eggs per fly; 1 week TRF = 3 eggs per fly; 2 weeks TRF = 8 eggs per fly; 4 weeks TRF = 8 eggs per fly). Thus, the flies' fecundity was enhanced and extended in response to 2 and 4 weeks of TRF.

Remarkably, we observed lifespan extension and increased fecundity following TRF. While lifespan extension linked to nutritional restrictions has been associated with reduced fecundity, this suggests that TRF‐specific changes lead to increased lifespan without compromising fecundity. In the next steps, we aimed to capture these changes in response to TRF across different cellular, physiological, and behavioral levels.

### 3.3 TRF Alters Body Composition Without Affecting Starvation Resistance Sustainably

**FIGURE 3:**
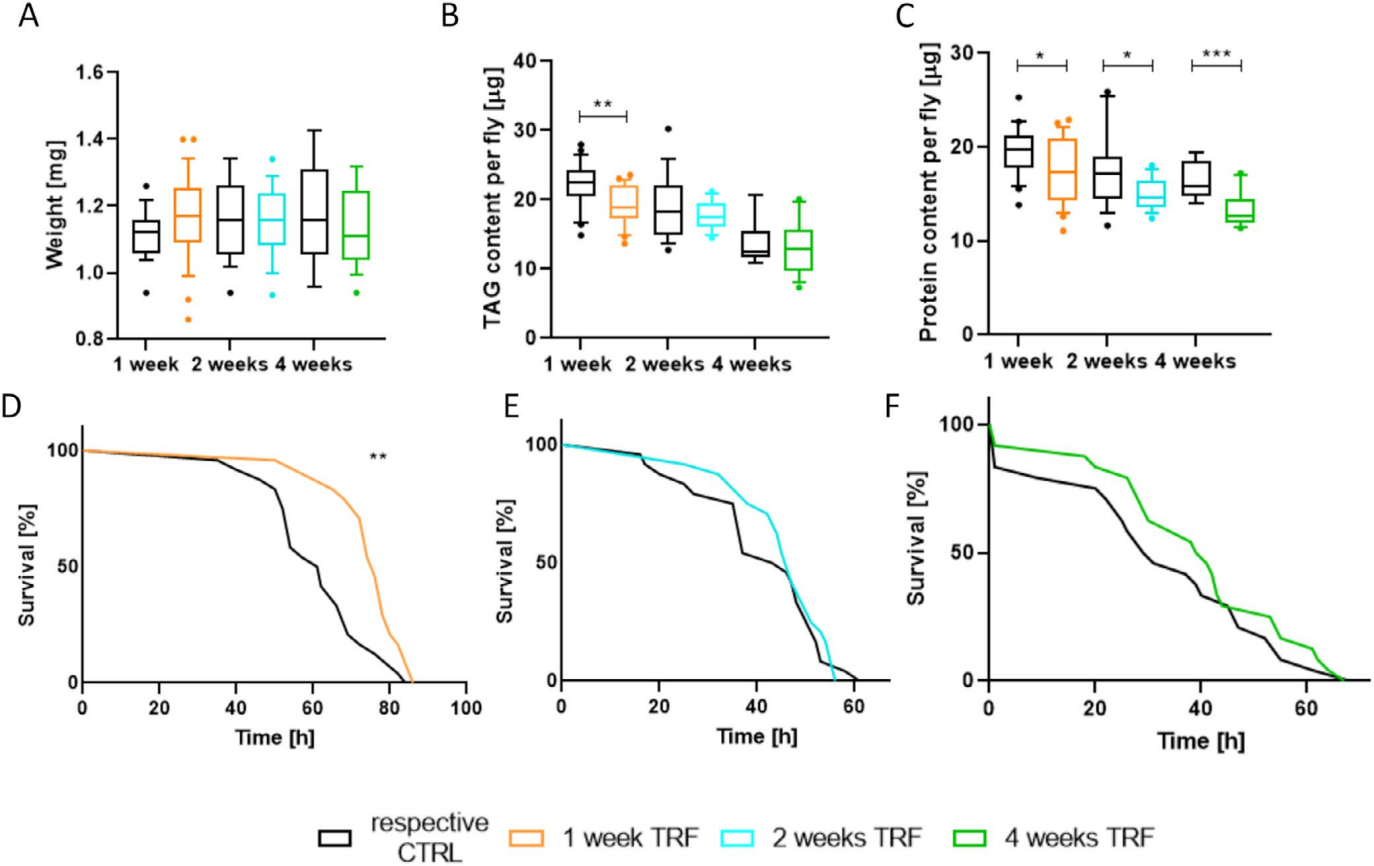
Body composition and starvation resistance in response to time‐restricted feeding in different intensities compared to control groups with continuous *ad libitum* access. (A) Bodyweight. (B) Lipid content. (C) Protein content. (D) Starvation resistance after 1 week of time‐restricted feeding. (E) Starvation resistance after 2 weeks of time‐restricted feeding. (F) Starvation resistance after 4 weeks of time‐restricted feeding. *n* = 9–25, *p* < 0.05*, *p* < 0.01**, *p* < 0.001***. Box and whiskers represent the mean and 10–90 percentile.

Energy metabolism and body composition are generally affected by nutritional interventions. Thus, we assessed the body composition of the flies after various durations of time‐restricted feeding (TRF) and compared it to their respective controls. Body weight exhibited no significant differences compared to the control group after 1, 2, or 4 weeks of TRF (Figure 3A). The lipid content significantly decreased after 1 week of TRF, with a mean lipid content per fly of CTRL = 22.1 μg/fly versus 1‐week TRF = 19.2 μg/fly (Figure 3B). However, this reduction in fat content diminished after 2 and 4 weeks of TRF, with mean values of CTRL 2 weeks = 18.79 μg/fly, CTRL 4 weeks = 13.95 μg/fly, 2 weeks TRF = 17.99 μg/fly, and 4 weeks TRF = 13.09 μg/fly. Notably, the lipid content per fly decreased with age. Furthermore, protein content was significantly influenced by TRF. The protein content per fly notably decreased in response to TRF after 1, 2, and 4 weeks compared to each respective control (mean: CTRL 1 week = 19.4 μg/fly, CTRL 2 weeks = 17.3 μg/fly, CTRL 4 weeks = 16.53 μg/fly, 1 week TRF = 17.4 μg/fly, 2 weeks TRF = 15 μg/fly, 4 weeks TRF = 13.39 μg/fly) (Figure 3C).

As we observed a significant shift in body composition in response to TRF, we evaluated the resistance to starvation after different periods of TRF (Figure 3D–F). After 1 week of TRF, starvation resistance increased significantly from 61.5 h in controls to 75.5 h in the experimental animals (Figure 3D). However, no notable change in the ability to withstand starvation was seen between the control group and the 2‐ and 4‐week TRF groups (Figure 3E,F).

### 3.4 TRF Reduces Food Intake Significantly but Does Not Affect Intrinsic Locomotion

**FIGURE 4:**
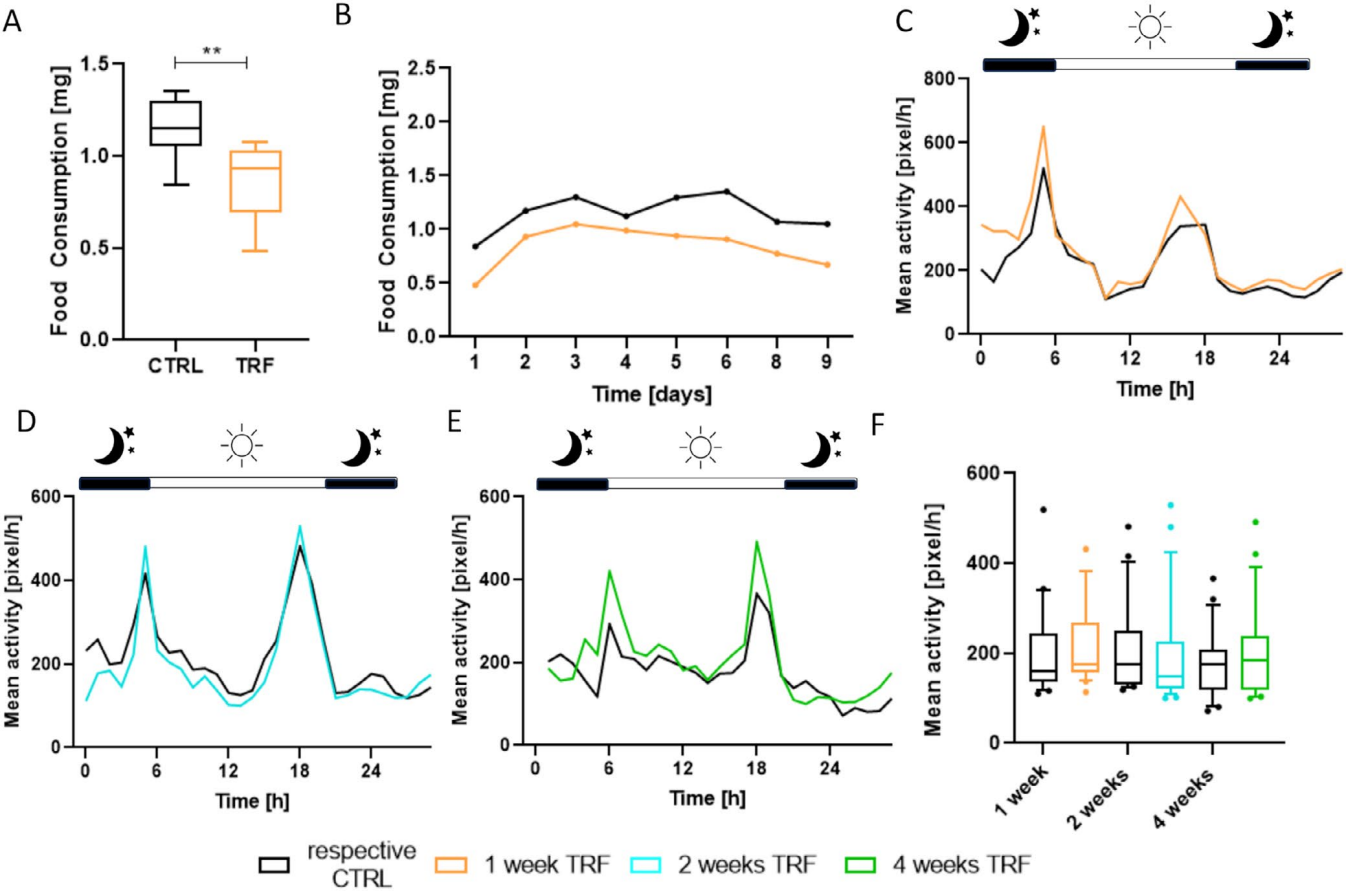
Daily locomotion pattern, average activity per hour, and food consumption after subjection to time‐restricted feeding for different durations compared to a relative control with continuous *ad libitum* access. (A) Locomotion pattern after subjection to 1 week of time‐restricted feeding. (B) Locomotion pattern after subjection to 2 weeks of time‐restricted feeding. (C) Locomotion pattern after subjection to 4 weeks of time‐restricted feeding. (D) Average Locomotion per hour. (E) Cumulative food consumption. (F) Daily average food consumption. *n* = 18–24, *p* < 0.01**. Box and whiskers represent the mean and 10–90 percentile.

As we observed changes in body composition, we wondered whether energy metabolism was also altered. Therefore, we first quantified food intake as the sole means of achieving energy intake. Due to the significant reduction in daily potential food intake imposed by the applied TRF conditions, we measured cumulative food consumption and conducted daily comparisons between TRF and control flies over 9 consecutive days (Figure 4A,B). This revealed a significant reduction in cumulative food consumption in response to 1 week of TRF (mean: CTRL = 1.15 mg/day, TRF = 0.84 mg/day). These results were consistent with the daily representation of food consumption, as the flies indeed consumed less food each day during the measurement period (Figure 4B). Thus, flies subjected to TRF exhibited reduced energy intake. Next, we quantified the daily locomotory profiles of the flies (Figure 4C–F). The activity profiles displayed the typical morning and evening peaks of maximum activity characteristic of the daily patterns of the flies. More specifically, a close resemblance was observed between each TRF group and their respective controls (Figure 4C–E), particularly after 1 and 2 weeks of TRF. However, after 4 weeks of TRF (Figure 4E), the control group exhibited age‐associated changes in movement patterns, with reduced morning and evening peaks. In contrast, the TRF‐treated animals did not show these age‐associated changes in movement patterns, although the differences in mean activities did not reach statistical significance (Figure 4F). Additionally, neither 1 nor 2 weeks of TRF significantly altered intrinsic average locomotion.

### 3.5 TRF Reduces Intestinal Stress and Preserves Gut Integrity

**FIGURE 5:**
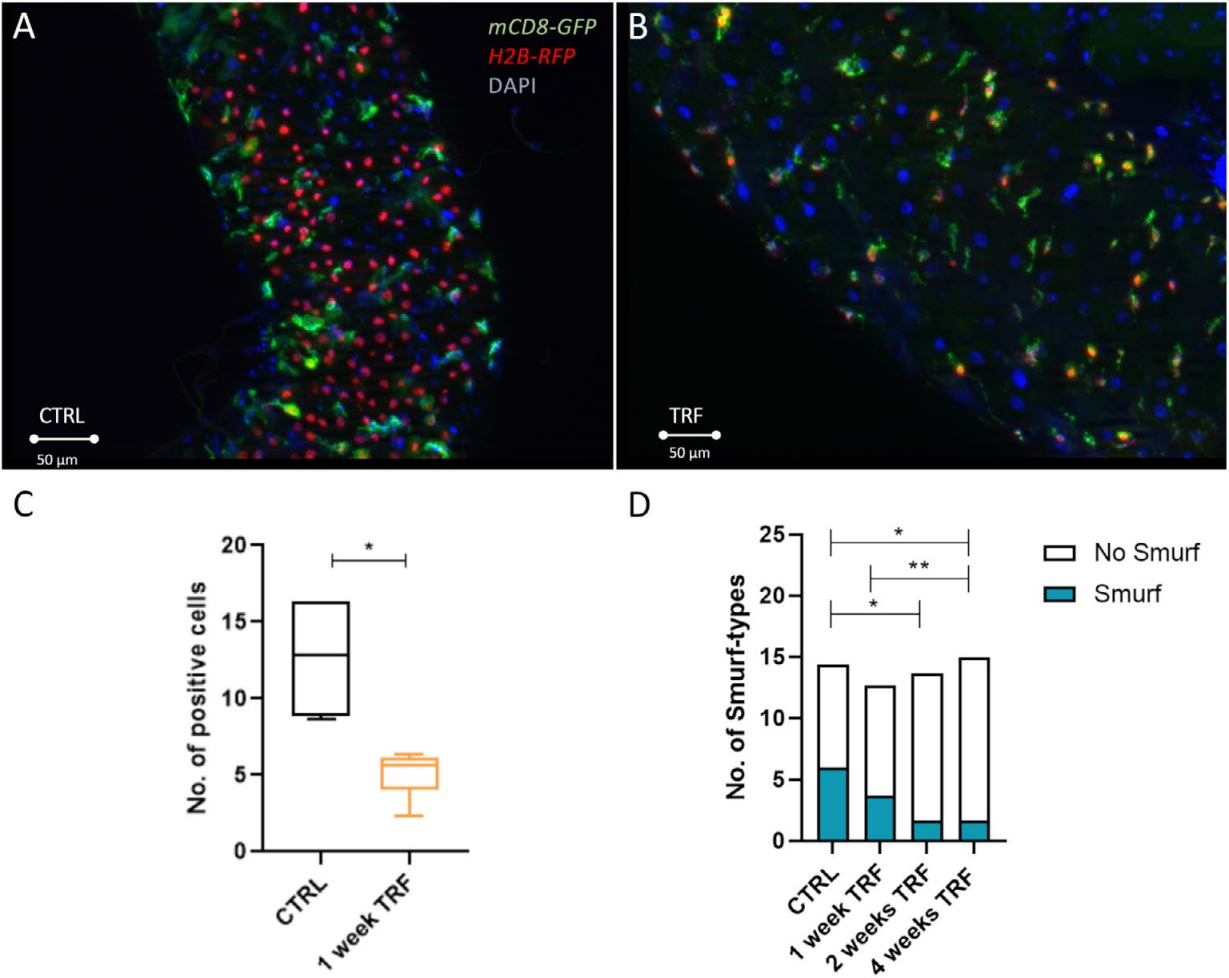
Dissected guts after subjection to 1 week of time‐restricted feeding, quantification of signals, and gut integrity measurements after time‐restricted feeding in different intensities. (A) Dissected gut of the control group. (B) Dissected gut after 1 week of time‐restricted feeding. (C) Quantification of newly differentiated cells based on several fluorescent signals in randomly assigned regions of interest. (D) After time‐restricted feeding in different intensities, gut integrity measurements are based on a ‘smurf’ assay. *n* = 8–15, *p* < 0.05*, *p* < 0.01**. Box and whiskers represent the mean and 10–90 percentile.

We focused on the aspects of intestinal physiology to identify possible mechanisms responsible for TRF's life‐prolonging effects. Age‐related changes in intestinal physiology are causally linked to reduced life expectancy. This is particularly evident in the age‐related increase in the proliferation rate of intestinal stem cells and the heightened permeability of the intestinal epithelium. For microscopic analysis of the fly's intestinal epithelium, we used a repressible dual differential stability cell marker (ReDDM) containing a fly line to visualize intestinal stem cell proliferation. Specifically, red nuclei indicate newly differentiated enterocytes (EC) and enteroendocrine cells (EE), while green signals represent freshly developed, undifferentiated ISCs, with the green signal decreasing as differentiation occurs. After hatching and mating, the flies were moved to 29°C to induce the expression system through heat. Following 1 week of either TRF or *ad libitum* feeding, the guts were dissected, counterstained with DAPI overlaid with the red signal, and analyzed using fluorescence microscopy (Figure 5A–C). Based on randomly selected regions of the intestine, we assessed the proliferation and turnover of stem cells as the mean number of red signals per region, which was quantified (Figure 5C). There was a significant reduction in proliferating ISCs, evidenced by a 53% decrease in the mean number of red signals in response to TRF (mean: CTRL = 13 red signals; TRF = 6 red signals; Figure 5A–C). Rera and colleagues [40] showed that intestinal integrity diminished with aging, resulting in ingested food being dispersed in the body cavity. The transfer of intestinal contents into the body cavity was tracked using blue food coloring. Here, animals with a blue‐colored abdomen that lost gut integrity were classified as the ‘Smurf’ type. The flies' gut integrity was assessed after 4 weeks under experimental conditions with varying durations of TRF and subsequent *ad libitum* feeding (Figure 5C). We found that 2 and 4 weeks of TRF significantly decreased the likelihood of increased gut permeability compared to the dietary control group (mean: CTRL = 6.3; 1 week TRF = 3.6; 2 weeks TRF = 1.6; 4 weeks TRF = 1.6 number of smurf types).

### 3.6 TRF Impacts the Microbiota Composition, and Infection With the Intracellular Symbiont Wolbachia Affects the Metabolic Reaction to This Intervention

**FIGURE 6:**
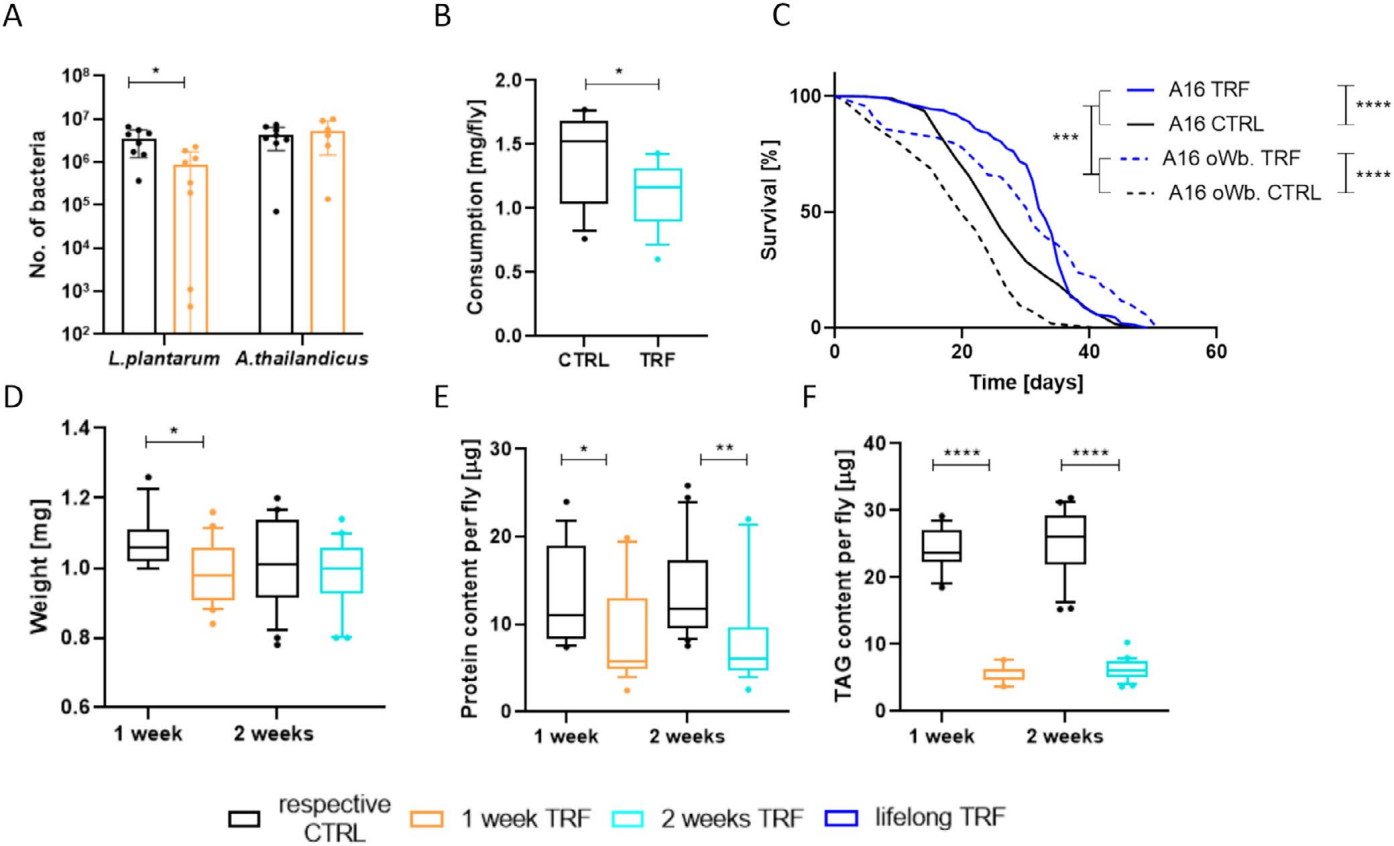
Bacterial establishment in the gut after 1 week of time‐restricted feeding and effects of time‐restricted feeding in *Wolbachia*‐free flies. (A) Abundances of *Lactobacillus plantarum* and *Acetobacter thailandicus* after 1 week of time‐restricted feeding compared to a control group with *ad libitum* access. (B) Lifespan of *Wolbachia*‐free flies subject to lifelong time‐restricted feeding compared to *Wolbachia*‐colonized flies and respective controls with continuous *ad libitum* access. (C) Cumulative food consumption of *Wolbachia‐*free flies after 2 weeks of time‐restricted feeding. (D) Bodyweight. (E) Lipid content. (F) Protein content. *n* = 8–200, *p* < 0.05*, *p* < 0.01**, *p* < 0.001***, *p* < 0.0001****. Box and whiskers represent the mean and 10–90 percentile.

The abundances of microorganisms colonizing the host intestinal tract were observed to underlie diurnal oscillations that are functionally linked to the feeding behavior of the host, resulting in time‐specific compositional profiles of the microbiome influenced by feeding patterns throughout the day. Therefore, we recolonized germ‐free flies with a 1:1 mix of two commensal bacterial species, *Lactobacillus plantarum* and *Acetobacter thailandicus*. TRF was administered for 1 week under sterile conditions to minimize the risk of contamination with other bacteria. The bacterial load of TRF and control flies was then assessed, revealing a significant decrease in the abundance of *L. plantarum* by 24.7% for the TRF flies (Figure 6A; mean: CTRL = 3.4 × 10^6^; 1‐week TRF = 8.5 × 10^5^). In contrast, no significant change in the absolute numbers of *A. thailandicus* was observed (mean: CTRL = 4.1 × 10^7^; 1‐week TRF = 5.2 × 10^7^). Endosymbiotic *Wolbachia* has already been observed to affect female fecundity [41], strengthen antiviral protection [42], and modulate lifespan [43]. To further assess how/if *Wolbachia* modulates physiological traits under TRF, experiments addressing primary metabolism, such as lifespan, alterations of the body composition, and food consumption, were repeated with *Wolbachia*‐free flies (Figure 6B–F). Similar tendencies between *Wolbachia*‐colonized and *Wolbachia*‐free flies were observed. However, the effects of TRF appeared to be enhanced by *Wolbachia* absence since a lifespan elongation of 55% was detected in these animals (Figure 6B; median: CTRL = days, lifelong TRF = days). TRF flies also significantly prolonged the maximum life expectancy, which was not observed in *Wolbachia*‐colonized flies. Corresponding to *Wolbachia*‐colonized flies, lipid and protein content significantly decreased in *Wolbachia*‐free flies in response to TRF. Notably, the lipid content was drastically reduced in response to TRF, reflecting a crucial role for *Wolbachia* in metabolic processes such as regulating lipid storage. This was also reflected by body weight that, contrary to *Wolbachia*‐colonized flies, was significantly reduced after 1 week of TRF (Figure 6D).

## 4 Discussion

TRF has emerged as a promising alternative to other dietary interventions. Its positive health effects and high acceptance rate make it an auspicious nutritional intervention [44, 45]. Our *Drosophila* model demonstrated that a classical TRF of 8 h of *ad libitum* feeding followed by 16 h of starvation significantly increases the median lifespan but not the maximum. Notably, the positive effect on lifespan is evident even with short TRF phases of 2 or 4 weeks early in life, while a 1‐week TRF during the same developmental period is ineffective. The 4‐week TRF is significantly more effective than the 2‐week TRF, suggesting a time dependency in effectiveness. For both short periods of TRF, we observed higher maximal lifespans and increased median lifespans. This discrepancy in the prolongation of maximum life expectancy, which we observe only for temporary TRF applications, suggests an age‐dependent complexity. TRF always positively affects median and maximum life expectancy relatively early in life. In contrast, it tends to have adverse effects later in life, especially at very old ages, and may increase mortality. This strong age dependency has also been observed with protein‐restricted diets in humans, where continuation of this otherwise beneficial intervention has negative health consequences [46]. Nutritional interventions early in life, such as the TRF applied in this study, positively influence lifespan. Early methionine restriction leads to comparable effects, as observed in this project [47]. Similarly, early rapamycin treatment was beneficial in both *Drosophila* and mice, while treatments later in life were ineffective [48]. Thus, it is reasonable to speculate that an early window of opportunity allows lifespan‐prolonging interventions to become effective TRF, unlike other nutritional interventions that extend lifespan and do not reduce fitness, i.e., the number of offspring. This unique benefit of TRF, which breaks the typical relationship between lifespan extension and reduced fitness, is a significant advantage over other interventions. This typical relationship is often seen as a necessary evil with CR‐ or DR‐induced lifespan extension [49, 50]. However, there are exceptions to this rule, such as with methionine restriction [51]. Thus, the results of the current study and the observations made in response to methionine restriction clearly show that lifespan prolongation and fitness reduction are not necessarily two sides of a coin but can be disentangled by the optimal type of nutritional intervention.

Restrictive dietary interventions such as calorie restriction, protein restriction, or TRF all benefit longevity and health, but the underlying mechanisms appear different. Manipulation of mTOR, AKT, or FoxO signaling and increased autophagy have usually been identified as the primary targets of these interventions [52, 53]. However, TRF is fundamentally different from CR and PR, as evidenced by the fact that the TRF‐compatible form of CR led to an additive increase in lifespan [54, 55].

Body composition analysis indicated a slight reduction in fat and protein content in flies exposed to short‐term TRF. We observed reduced protein levels during extended TRF periods, while fat levels remained unchanged. These differences between the short‐term and long‐term effects of TRF suggest fundamental mechanistic differences between these two outcomes. In the short term, the primary factor is the energy derived from the use of both fat and protein stores. In contrast, the long‐term impact of TRF leads to a complex metabolic change that is not yet fully understood and particularly safeguards fat stores. Surprisingly, short‐term TRF enhanced starvation resistance, a response typically linked with increased fat stores. The reason behind this observation remains unclear. Based on these results, we also assessed energy expenditure and energy intake. TRF did not influence movement, the most critical aspect of energy expenditure. Interestingly, we noted a significant reduction in food intake, the sole source of energy. The decrease in overall energy intake diminishes fat and/or protein stores.

In addition to the metabolic changes triggered by periods of TRF that may contribute to a longer life, other factors also play a crucial role in lifespan. Intestinal physiology appears to be a key determinant of lifespan since aging intestines tend to become leaky, allowing the transfer of bacteria into the body cavity, which is typically lethal. Therefore, we examined the main factors influencing gut health and integrity, including the proliferative activity of intestinal stem cells and overall gut integrity. Notably, the basal proliferative activity is significantly lower following TRF than age‐matched controls, a characteristic of young, fully functional guts [56]. This reduction is also observed with caloric or protein restriction in both physiological and pathophysiological contexts [24, 57, 58]. Improvements in intestinal biology are also evident in the smurf assays, which help identify animals with a leaky gut, indicating organ aging. TRF significantly mitigated this detrimental phenotype, suggesting that enhanced intestinal health may contribute to longevity extension. The effects of TRF on the gut microbiota are directly related to the effects on gut health. We selected an engineered microbiota comprising one gram‐positive and one gram‐negative bacterium to study these outcomes. Our findings revealed that TRF specifically reduced the quantity of *Lactobacillus plantarum* , leading to a significant shift in the microbial community. Like other dietary interventions, TRF alters the composition and, presumably, the function of the intestinal microbiota [26, 59]. We also investigated the effects of the endosymbiont Wolbachia, a bacterium present in approximately 50% of all wild‐caught and laboratory strains that influence various aspects of the physiology and life history of the fly [60, 61, 62]. All our experiments were conducted using a strain containing Wolbachia. After depleting this strain of Wolbachia and culturing it for more than 10 generations to restore the normal microbiota, we subjected these Wolbachia‐free animals to detailed phenotyping. The results were similar to those of the Wolbachia‐infected animals but revealed intriguing differences. The TRF‐induced lifespan extension was more pronounced and included median and maximal lifespan. We also observed a more substantial effect, particularly on fat stores, which were drastically reduced in response to TRF, suggesting that *Wolbachia* dampens lifespan extension and alters body composition.

Overall, TRF and even brief periods of TRF resulted in significant changes in flies' physiology without affecting their fitness. The metabolic change‐induced modifications, particularly those in intestinal properties, may account for the health‐promoting benefits of this simple and easy‐to‐follow nutritional intervention. This further supports the application of TRF in humans, not only for weight loss but primarily for the associated health benefits. A cautionary note should be considered to understand *Wolbachia*'s role in these various manipulations.

## Author Contributions

A.‐C.H. was responsible for data acquisition, analysis, and drafting of the article; M.K. performed additional experiments; S.K.‐E. and T.R. wrote the final version and designed the study.

## Conflicts of Interest

The authors declare no conflicts of interest.

## Data Availability

This study did not generate new unique reagents.
